# Complement C5a Alters the Membrane Potential of Neutrophils during Hemorrhagic Shock

**DOI:** 10.1155/2018/2052356

**Published:** 2018-05-29

**Authors:** David A. C. Messerer, Stephanie Denk, Karl J. Föhr, Rebecca Halbgebauer, Christian K. Braun, Felix Hönes, Julia Harant, Michael Fauler, Manfred Frick, Benedikt L. Nußbaum, Peter Radermacher, Sebastian Hafner, Markus S. Huber-Lang

**Affiliations:** ^1^Institute of Clinical and Experimental Trauma-Immunology, University Hospital Ulm, 89081 Ulm, Germany; ^2^Department of Anesthesiology, University Hospital Ulm, 89081 Ulm, Germany; ^3^Institute of General Physiology, Ulm University, 89081 Ulm, Germany; ^4^Institute of Anesthesiologic Pathophysiology and Method Development, University Hospital Ulm, 89081 Ulm, Germany

## Abstract

**Background:**

Polymorphonuclear granulocytes (PMN) play a crucial role in host defense. Physiologically, exposure of PMN to the complement activation product C5a results in a protective response against pathogens, whereas in the case of systemic inflammation, excessive C5a substantially impairs neutrophil functions. To further elucidate the inability of PMN to properly respond to C5a, this study investigates the role of the cellular membrane potential of PMN in response to C5a.

**Methods:**

Electrophysiological changes in cellular and mitochondrial membrane potential and intracellular pH of PMN from human healthy volunteers were determined by flow cytometry after exposure to C5a. Furthermore, PMN from male Bretoncelles-Meishan-Willebrand cross-bred pigs before and three hours after severe hemorrhagic shock were analyzed for their electrophysiological response.

**Results:**

PMN showed a significant dose- and time-dependent depolarization in response to C5a with a strong response after one minute. The chemotactic peptide fMLP also evoked a significant shift in the membrane potential of PMN. Acidification of the cellular microenvironment significantly enhanced depolarization of PMN. In a clinically relevant model of porcine hemorrhagic shock, the C5a-induced changes in membrane potential of PMN were markedly diminished compared to healthy littermates. Overall, these membrane potential changes may contribute to PMN dysfunction in an inflammatory environment.

## 1. Introduction

Innate immunity fulfils a crucial role in host defense as part of the first line of defense against pathogens. In physiological settings, polymorphonuclear leukocytes (PMN) respond to chemoattractants such as activated complement factor 5 (C5a) with cellular migration, increased apoptotic resistance, and elimination of pathogens by generation of reactive oxygen species (ROS) via activation of the NADPH oxidase [1, 2].

Systemic inflammation during sepsis or traumatic-hemorrhagic shock releases a storm of damage- and pathogen-associated molecular patterns (DAMPs and PAMPs, resp.). Although not yet completely unraveled, this plays a role in generating and releasing incompetent neutrophils with a striking discrepancy between preserved morphological integrity and functional incompetence [3, 4]. In combination with hypoxia and acidosis, this immunological dysfunction may drive the host into infectious complications and multiorgan dysfunction syndrome (MODS) resulting in a devastating mortality [3].

The complement activation product C5a interacts not only with PMN via abundantly expressed specific receptors (C5aR1 = CD88 and C5aR2 = C5L2 = GPR77) [5, 6] but also with other immune cells and epithelial cells [6]. G-protein-mediated C5aR1 signaling results predominantly in the release of intracellular calcium [5–8] in PMN, which is likely triggered by changes of membrane electrophysiology as alterations in membrane potential, ion channel permeability, and fluxes. In this context, changes in membrane potential [9–11] and transient intracellular alkalinization [12–15] represent early hallmarks of PMN activation by fMLP or phorbol myristate acetate (PMA). In contrast, neutrophils in chronic granulomatous disease are characterized by a diminished production of ROS leading to the absence of cellular depolarization and an impaired immune response [9, 16]. There is a lot of evidence that PMA- or fMLP-driven initial depolarization of PMN is mediated by NADPH oxidase activity followed by a proton-driven compensation ([11, 15–28] and more; see [29, 30] for reviews). To define detailed electrophysiological features of the cell membrane including membrane potential, patch-clamp techniques are often applied. However, in the case of electrophysiological PMN characterization, the amount of studies is surprisingly limited. In one study, the resting membrane potential of PMN was proposed to be approximately −60 mV [31]. Generally, whole-cell patch-clamp approaches may provoke PMN activation by rupture of the cellular membrane and tampering with the intracellular milieu and induce futile phagocytic efforts of the glass pipette (own unpublished observations and [11, 23]). Albeit there are a few patch-clamp studies on granulocytes which provide valuable information, they can be associated with possible limitations as electrophysical measurements in neutrophils are difficult by nature, for example, description of NADPH oxidase of other cells (eosinophils [16, 19, 24], the granulocytic cell line HL-60 [21, 22], or macrophages [15]), application of intracellular milieu changing ionophores (e.g., valinomycin, gramicidin, and amphotericin) [11, 18, 23], or the use of enucleated neutrophils [17, 20]. To avoid these artificial impacts plus vastly increasing the number of measured cells, we characterized and applied a flow cytometry-based approach.

In the present study, we hypothesized that C5a may alter electrophysiological properties as membrane potential, intracellular pH, and mitochondrial membrane potential of PMN. Furthermore, we proposed that the described C5a-induced functional paralysis of PMN during systemic inflammation [32, 33] may be associated with impairment of electrophysiological features of the membrane of PMN.

## 2. Materials and Methods

Unless otherwise stated, all chemicals were obtained from Sigma-Aldrich (Darmstadt, Germany).

### 2.1. Isolation of Polymorphonuclear Neutrophils from Humans and Pigs

After approval by the Local Independent Ethics Committee of the University of Ulm (number 244/11 and 94/14) and written informed consent was collected, human blood was drawn by peripheral venous puncture in sodium citrate monovettes. Subjects were healthy males and females between 18 and 35 years without signs of infection or any current medical problems or medication.

Animal experiments were approved by the Federal Authorities for Animal Research, Tübingen, Germany (number 1087), in accordance to the ARRIVE guidelines. Male Bretoncelles-Meishan-Willebrand cross-bred pigs (median age: 14.5 months, interquartile range: 13 to 16 months; median weight: 68 kg, interquartile range: 61 to 73 kg) were purchased from Laboratoire de Thrombose et d'Athérosclérose, Institut des Vaisseaux et du Sang, Hôpital Lariboisière, Paris, France. Animals were sheltered at Oberberghof, Ulm, Germany, until further use. Animals were kept at a cycle of 12/12 hr light/darkness and were at least monitored daily.

In brief, hemorrhagic shock was achieved by removal of 30% of the blood volume followed by titration of the mean arterial pressure at 40 mmHg for a period of 3 hr. Samples were taken before and at the end of the 3 hr shock period via a carotid arterial line. To reduce experimental animal numbers, the samples of interest were taken from a subgroup (before further treatment) of the recently published porcine hemorrhagic shock study which provides the detailed protocol including anesthesia, pre- and perioperative fluid management, and the complex instrumentation procedures [34, 35]. After further interventions (which all occurred after the blood drawing relevant to this study), animals were euthanized under deep anesthesia by injection of potassium chloride. We chose hemorrhagic shock as a DAMP-driven and well-standardized model of significant inflammation as it is accompanied by severe systemic PMN activation [3, 36]. Moreover, it is less likely that there are any potentially influencing effects of external factors such as PAMPs released in sepsis models.

In human and similarly in pigs, PMN were isolated by Ficoll-Hypaque gradient centrifugation and dextran sedimentation. After hypotonic lysis of remaining erythrocytes, cell concentration was adjusted at 2 × 10^6^ per milliliter. Cells were suspended in K4.5 for further use.

### 2.2. Preparations of Buffers

Buffers were prepared with different levels of sodium (Na^+^) and potassium (K^+^) with a total amount of 145.4 mmol/l (mM). Buffers were labeled according to their K^+^ concentration; for example, buffer K4.5 contains 4.5 mM K^+^ and 140.9 mM Na^+^. Furthermore, buffers contained 1 mM MgCl_2_, 1 mM CaCl_2_, 5 mM glucose, and 10 mM HEPES. Finally, buffers were adjusted to a pH of 7.4 unless indicated otherwise. Preparations were controlled by a blood gas analysis device (model ABL 700, Radiometer GmbH, Willich, Germany) and an osmometer (Fa Gonotec, Berlin, Germany).

### 2.3. Measurement of Membrane Potential

**Figure 1:**
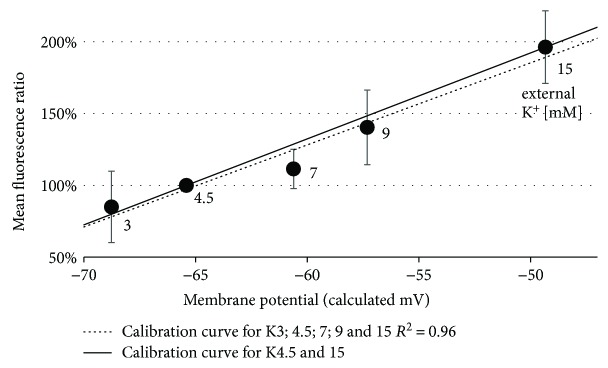
Calibration curves for alterations in the mean fluorescence depending on the calculated membrane potential. Shown here are the linear regression curves for two (solid line) or five different potassium concentrations (dotted line). 4.5 mM potassium was considered the physiological extracellular concentration and set to 100%. The method allows the detection of small alterations in membrane potential with an increase in the fluorescence of 6.0% (solid line) or 5.7% (dotted line) per mV increase in membrane potential. *N* = 5 per concentration.

Isolated PMN were incubated with 40 *μ*M bis(1,3-dibutylbarbituric acid) trimethine oxonol (DiBAC_4_(3)) for 10 min for measuring the membrane potential. The Goldman-Hodgkin-Katz (GHK) equation [37, 38] was used in a simplified version to approximate the cellular membrane potential (Supplement 1 Equation 1). Intracellular Na^+^ and K^+^ levels were assumed to be 14 mM and 140 mM. Relative transmembrane permeability (P) was assumed to be 5 : 95 for Na^+^ : K^+^. As other ions remained constant in the buffers, they were not taken into account. In total, for K4.5 buffer, membrane potential was calculated to be −65.4 mV (K15 = −49.3 mV). The resulting slope (Supplement 1 Equation 2) was used to quantify the amount of change in membrane potential (Supplement 1 Equation 3). To enhance the reliability of the results, a control (Ctrl) for each probe and time point with K4.5 buffer and depolarized cells with K15 buffer was measured (Figure 1). All values are presented as changes in membrane potential compared to control cells, as absolute values of membrane potential could not be obtained reliably and are based on the described calculations.

### 2.4. Measurement of Intracellular pH

Isolated PMN were preloaded with 1 *μ*M seminaphtharhodafluor (SNARF) for 20 min for measuring the intracellular pH. Cells were washed once before measurement. Internal pH was determined using a standard curve applying nigericin as indicated by the manufacturer and described previously [14]. In experiments, where membrane potential and internal pH were synchronically assessed, SNARF was added for 20 min following 10 min of incubation with DiBAC_4_(3).

### 2.5. Measurement of Mitochondrial Membrane Potential

To determine the mitochondrial membrane potential, PMN were incubated with 200 nM 5,5′,6,6′-tetrachloro-1,1′,3,3′-tetraethyl-imidacarbocyanine iodide (JC-1) for 30 min. Mitochondrial membrane potential was compared with cells whose mitochondria were treated with the uncoupler carbonyl cyanide 3-chlorophenylhydrazone (CCCP) at 50 *μ*M.

### 2.6. Stimulation of PMN and Flow Cytometry Analysis

Cells were stored in a darkened water bath at 37°C and incubated with the respective dye as described. Stimulants (e.g., human recombinant C5a, if not indicated otherwise in a concentration of 100 ng/ml) were added directly after the dye incubation period. Usually, 10000 cells were measured per sample (5000 at least) using BD FACSCanto II (Franklin Lakes, USA) as flow cytometer. PMN cell population was analyzed by setting a proper gate using a side-scattered light area (SSC-A) and a forward-scattered light area (FSC-A) followed by a removal of duplets (usually <2%). In experiments with subgroup analysis of neutrophils, additional gates were applied to examine separately the 25% PMN with the highest and the 25% with the lowest SSC/FSC values, respectively. Measurements of CD88 (C5aR1) were conducted after incubating cells with FITC-labeled anti-CD88 antibody for twenty minutes. The results are shown as mean fluorescence after subtraction of an appropriate isotype control.

### 2.7. Statistical Analyses

Statistical analyses were performed using GraphPad Prism 5 (GraphPad Software Inc., USA). The data were expressed as mean ± standard deviation (SD). Outliers were identified and purged by using the *z*-score test. Gaussian distribution was determined by D'Agostino-Pearson omnibus normality test. In case of a two-group comparison, the unpaired *t*-test for normal distribution and the Mann–Whitney *U* test for non-Gaussian distribution were applied. If comparison versus zero was of interest, the *t*-sample *t*-test (for normality) and the Wilcoxon signed-rank test (for nonnormality) were applied. For multiple comparisons, ANOVA with Tukey post hoc testing was used (normality) or Kruskal-Wallis analysis with Dunn's post hoc testing (nonnormality). The preset significance levels of *p* < 0.05, <0.01, and <0.001 are shown by ∗, ∗∗, and ∗∗∗, respectively. Stars without link to another group express significance versus zero or control.

## 3. Results

### 3.1. C5a Depolarizes PMN in a Concentration- and Time-Dependent Manner

**Figure 2:**
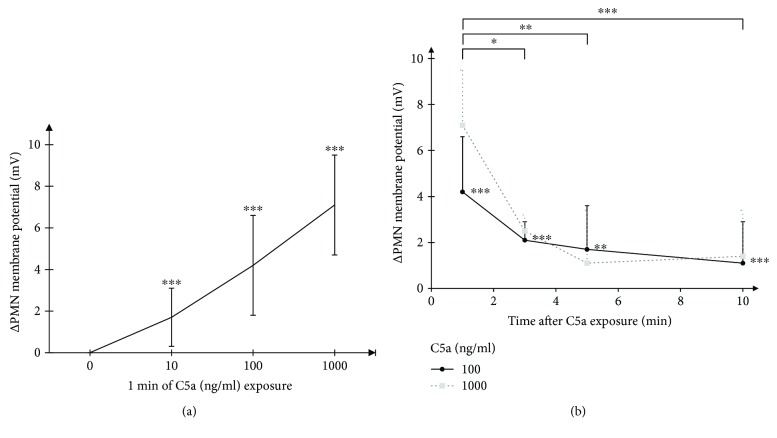
(a) Concentration dependency. Concentration-dependent change in resting membrane potential in human neutrophils after incubation with C5a demonstrating a depolarization in a dose-dependent manner (*n* = 11; 45; 6). (b) Time dependency. Time-dependent change of resting membrane potential of human PMN after stimulation with C5a 100 ng/ml (straight black line) and C5a 1000 ng/ml (gray dotted line), *n* = 3–45. All significance indicators refer to C5a 100 ng/ml. ^∗^ *p* < 0.05, ^∗∗^ *p* < 0.01, and ^∗∗∗^ *p* < 0.001.

The described method was internally validated using different buffers (Figure 1). After stimulation with C5a in various concentrations (conversion 100 ng/ml ≈ 9.1 nM), PMN of healthy human donors responded with a significant shift in membrane potential towards depolarization (Figure 2(a)). The early C5a-induced depolarization declined within 10 min at both C5a concentrations, 100 ng/ml and 1000 ng/ml (Figure 2(b)).

### 3.2. C5a Invokes a Change in Cell Shape Independent of Membrane Potential Changes

**Figure 3:**
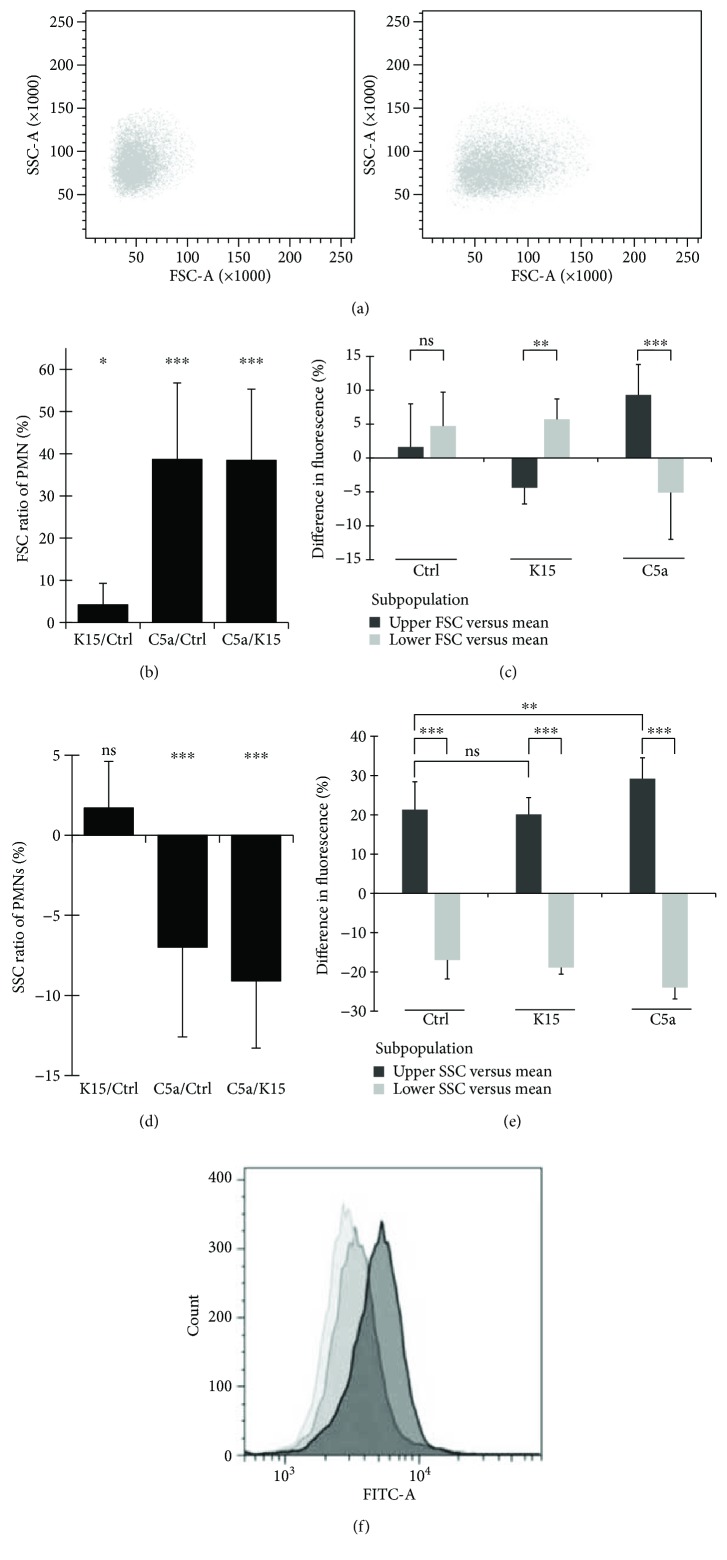
(a) Change in the shape and granularity of PMN after C5a stimulation. Influence of 1 minute C5a 100 ng/ml exposure on SSC-A (indicator for granularity) and FSC-A (indicator for changes in cell shape) on human PMN. (b) C5a-induced change in cell shape cannot be achieved by forced depolarization. Change in FSC-A for human PMN (*n* = 10–12) after incubation with 10 minutes in buffers plus 1 minute of C5a 100 ng/ml. (c) Independency of cell shape from membrane potential. Difference of membrane potential in human PMN subgroups (*n* = 10–12). Shown is the relation between the 25% upper and lower part of cells in relation to the average fluorescence. (d) C5a-induced change in cell granularity cannot be achieved by forced depolarization. Change in SSC-A for human PMN (*n* = 10–12) after incubation with 10 minutes in buffer plus 1 minute of C5a 100 ng/ml exposure = 11 minutes in total. (e) Independency of cell granularity from membrane potential. Difference of membrane potential in human PMN subgroups (*n* = 10–12). Shown is the relation between the 25% upper and lower part of cells in relation to the average fluorescence. (f) Representative illustration of determination of membrane potential. DiBAC_4_(3)'s fluorescence was measured in the FITC channel. Each sample consists of Ctrl (PMN in K4.5 = light gray), PMN exposed to C5a 100 ng/ml in K4.5 for one minute (gray), and PMN incubated in K15 (dark gray). ^∗^ *p* < 0.05, ^∗∗^ *p* < 0.01, and ^∗∗∗^ *p* < 0.001.

The chemoattractant C5a has an impact on cellular shape [39]. Therefore, FSC-A values as indicators for cell shape and SSC-A values as indicators for cellular granulation were monitored to investigate whether subgroups of PMN react differently and to exclude that the change in fluorescence intensity indicating depolarization was caused by changes in SSC and FSC recording. Cell shape as indicated by FSC-A increased in response to C5a 100 ng/ml by 39%, while the depolarization by K15 was not able to provoke a similar response (Figures 3(a) and 3(b)). Subgroups of PMN (upper versus lower 25% according to FSC-A measurements) did not show a potential difference to control cells. However, after stimulation with C5a, cells with higher FSC-A value exposed a significantly stronger fluorescence signal indicating an increased shift in membrane potential (Figure 3(c)). C5a also mediated a change in cellular granularity, resulting in a small but significant decrease by 7% in SSC-A. Again, this effect could not be observed when using potassium to depolarize the cells (Figure 3(d)). Nonetheless, cells with higher granularity expressed increased fluorescence intensity. This effect was augmented by C5a stimulation. In addition, a “forced” depolarization applying higher extracellular potassium concentrations did not explain the changes in fluorescence intensity, suggesting that the used dye was not (SSC) or only marginally (FSC) effected by cell shape changes (Figure 3(e)). Control cells, stimulated PMN with C5a 100 ng/ml, and depolarized PMN remained in one population (Figure 3(f)). Therefore, a shift in mean fluorescence of stimulated PMN is mediated by a fluorescence increase in general, not by an extreme shift of a minor subgroup.

### 3.3. Interaction of the Cellular Membrane Potential with the Internal and External pH

**Figure 4:**
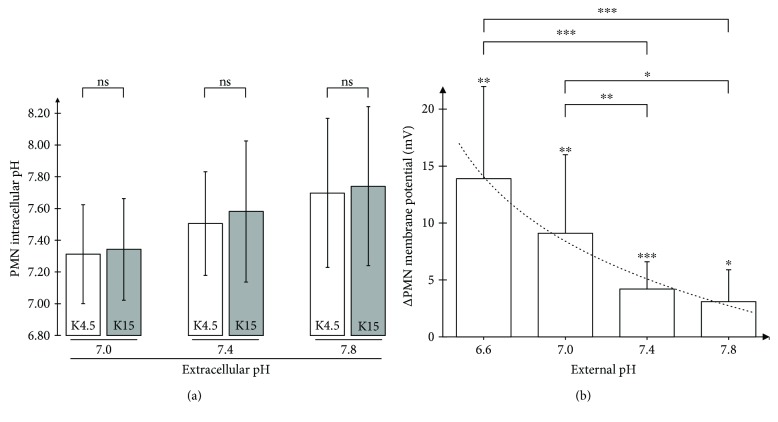
(a) Independency of intracellular pH from forced depolarization. PMN with different external pH, each with K4.5 (gray column) and K15 (white column) after a 10-minute incubation (*n* = 5). (b) Dependency of extracellular pH and response to C5a. Comparison of different external pH influence on the change of membrane potential of human PMN after stimulation with C5a 100 ng/ml after 1 minute; *n* = 8; 10; 45; 9. The dotted line represents a logarithmic trend line (*R* ^2^ = 0.98). ^∗^ *p* < 0.05, ^∗∗^ *p* < 0.01, and ^∗∗∗^ *p* < 0.001.

A change in cellular membrane potential and external pH represents a major impact on the driving force of protons. Therefore, we investigated whether a change in membrane potential and external pH subsequently influences intracellular pH. There was some evidence of an interconnection between the intracellular and extracellular pH. However, there was no significant difference comparing control cells with depolarized cells by K15 (Figure 4(a)).

Inflammatory processes are often accompanied by extracellular acidification of the (micro)environment. As depicted in Figure 4(b), the initial response of the membrane potential of human PMN to C5a significantly increased with decreased external pH values. However, after 10 min, the response of the membrane potential returned to values of unstimulated cells (data not shown).

### 3.4. Mitochondrial Membrane Potential Appears Resistant against C5a Exposure

**Figure 5:**
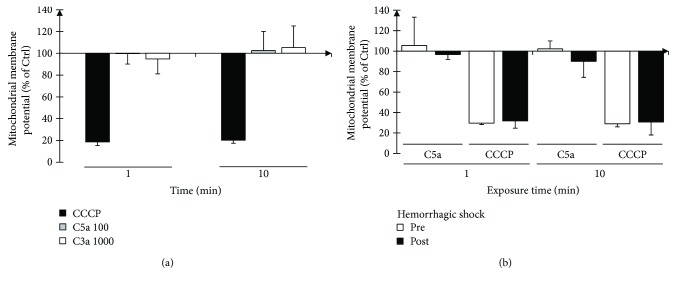
(a) Measurement of mitochondrial membrane potential of human PMN. No significant change in mitochondrial membrane potential occurred when human PMN are stimulated with C5a or C3a. In contrast, the potent uncoupler CCCP can effectively alter the mitochondrial membrane potential (*n* = 6; 5; 4). (b) Mitochondrial membrane potential before and after hemorrhagic shock. While C5a does not alter the mitochondrial membrane potential, the breakdown conjured by CCCP remains nearly the same (*n* = 3-4).

To further evaluate electrophysiological features of PMN and to determine whether C5a-induced delay in apoptosis during systemic inflammatory conditions (such as during shock) [40] is linked to changes in mitochondrial membrane potential, its reaction towards C5a exposure was investigated. Mitochondrial membrane potential was responsive to uncoupling. However, there was no significant alteration of the mitochondrial membrane potential by the central anaphylatoxins, neither to C5a nor to C3a (Figure 5(a)). Mitochondrial membrane potential appeared also resistant to C5a exposure in porcine PMN before and after experimental severe shock (Figure 5(b)). Moreover, LPS challenge as an example of PAMP stimulation failed to alter mitochondrial membrane potential of PMN (data not shown).

### 3.5. The Chemoattractant fMLP but Not C3a Reveals Effects Similar to C5a

**Figure 6:**
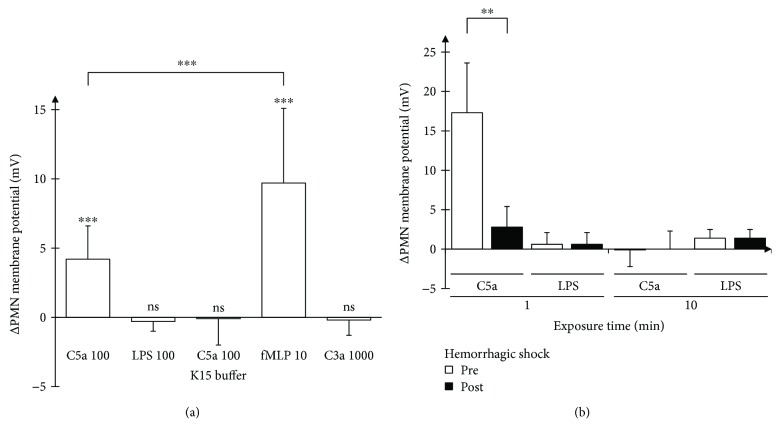
(a) Assessment of further stimulants. Change in membrane potential of human PMN when exposed to different substances (each in ng/ml, expect fMLP in *μ*M, *n* = 45; 6; 8; 10; 3). (b) Membrane potential of PMN in hemorrhagic shock. Change in membrane potential of porcine PMN before and after hemorrhagic shock (C5a or LPS 100 ng/ml, *n* = 4; 5). ^∗∗^ *p* < 0.01 and ^∗∗∗^ *p* < 0.001.

For further characterization, we compared the effects of different potential chemotactic stimuli on the membrane potential shift in human PMN (Figure 6(a)). The central complement factor C3a (1000 ng/ml) and LPS (100 ng/ml) both failed to alter the membrane potential of human PMN. In contrast, fMLP generated an even stronger response than C5a in altering the membrane potential. As for stimulation with C5a (Figure 3(f)), PMN depolarization distributed almost homogenously among the cells and was not driven by an extreme depolarization of certain subgroups (data not shown).

### 3.6. Predepolarization Abolishes the C5a-Caused Membrane Potential Alterations

As a next step, we tested whether a prior depolarization influences the reaction of PMN to C5a. PMN suspended in K15 thus being depolarized by ~15 mV for 10 min showed no reaction to C5a after 1 min in stark contrast to normal cells (Figure 6(a)). We therefore examined whether a depolarization by K15 leads to a change in C5aR1 expression, as assessed by flow cytometry 20 min after C5a exposure. In this setting, no significant effect on C5aR1 (=CD88) surface expression could be detected (Table 1).

### 3.7. Breakdown of Membrane Potential Response to C5a in Hemorrhagic Shock

After characterization of the response in healthy subjects, we aimed to investigate the change under severe pathophysiological conditions. We chose a model of pig hemorrhagic shock and compared the response of porcine PMN to C5a and LPS. Prior to hemorrhage, porcine PMN responded to C5a in a markedly stronger fashion than did human PMN (Figure 6(b)). However, this response was markedly reduced at the end of the shock period. The time-course of the membrane potential in response to C5a was roughly comparable to the human conditions. Of note, the membrane potential of PMN did not feature a response to LPS neither pre- nor post-hemorrhagic shock. There was no change in potassium in the plasma samples of the animals (Table 2) and, as a consequence, no change in the calculated resting membrane potential prior and past hemorrhagic shock. However, the blood was diluted to some extent as indicated by the values of hemoglobin. C5a plasma preshock concentrations (10.3 ± 3.3 ng/ml) were similar to post-shock concentrations (10.5 ± 0.9 ng/ml) excluding a principal receptor downregulation by excessive C5a. At last, we assessed whether there were any endotoxins present in the circulation pre- or posthemorrhagic shock. However, endotoxin concentrations were below the detection limit in all samples (data not shown), excluding LPS preexposure effects.

## 4. Discussion

### 4.1. Overview

Significant depolarization of the membrane potential of PMN could be found in a dose-dependent manner after one minute of C5a exposure. C5a in concentrations between 100 and 1000 ng/ml induced a transient depolarization that decreased markedly until ten minutes. The applied method allowed generation of precise calibration curves between one and ten minutes. Therefore, we might have missed very early and late changes in membrane potential differences, for example, a theoretical early higher maximum at C5a 1000 ng/ml. The used C5a concentrations may be considered within the range of (patho)physiological concentrations (EC50 C5a on C5aR1 ≈ 11 ng/ml) [5]. C5a concentration varies in physiological conditions around 0–20 ng/ml (110 ng/ml in one report), increasing to 100–1000 ng/ml in sepsis, and was reported to reach up to 3100 ng/ml after activation with cobra venom factor [5, 6, 41, 42]. Here, we suggest that the electrophysiological consequences of PMN membrane potential may depend on different C5a concentrations. While it was not within the scope of the present study to determine the responsible ion flux, it might be linked to activation of NADPH oxidase [27, 28, 30] and/or a change in potassium conductance, as PMN possess several potassium channels [31]. The major role of potassium conductance for transmembrane potential in human PMN is supported by studies using other fluorescence dyes in the presence or absence of the K^+^ ionophore valinomycin [9, 43, 44].

Analyzing subgroups of PMN, we demonstrated that C5a changes cell shape (Figure 3(a)) and leads to a more pronounced transmembrane depolarization in larger cells as characterized via higher FSC-A values. Forced depolarization did not trigger reduction in cellular granularity. However, C5a reduced the level of granularity, and cells with higher granularity emitted in the resting as well as in stimulated state a higher level of fluorescence indicating a more positive transmembrane potential. Of interest, we did not have any hint that degranulation does result in significant loss of dye. Even if this is the case, we might have slightly underestimated the change in membrane potential, as an increase in fluorescence indicates depolarization. While we cannot completely exclude that this is due to a specific dye characteristic, the different levels of granularity likely represent PMN of different age.

Surprisingly, forced depolarization by extracellular K15 had no effect on intracellular pH. Theoretically, the inward proton-driving force should be reduced by a positive shift in the cellular membrane potential (Figure 4(a)). It is tempting to speculate that resting PMN have only a small conductance for protons or that intracellular pH is balanced. In addition, the early transmembrane potential response to C5a was enhanced in external acidification conditions that occur during excessive inflammation and are known to promote MODS. Interestingly, another study using PMA to stimulate neutrophils described reduced depolarization at external pH values of 6.6 [17], possibly due to different compensation mechanisms depending on the initial stimulus.

In a translational model of porcine hemorrhage, shock led to neutrophil unresponsiveness to C5a. As neutrophil activation during the shock period cannot be excluded, the impaired response of neutrophils towards C5a underlines the role of an altered response of transmembrane potential as part of the pathophysiological reaction of PMN in severe inflammation. Although human recombinant C5a was used to stimulate porcine PMN, it is chemically similar to porcine C5a [5] and was capable to evoke a significant membrane potential response prior to hemorrhagic shock, which was more pronounced than in the human in vitro setting.

### 4.2. Strengths of the Study

We established and validated a reliable method for PMN to detect even small changes in transmembrane potential towards hyper- or depolarization (Figure 1), which has been previously described for other cell types and controlled by patch-clamp technique [38, 45]. As the slope of the calibration curve remained stable over a certain range of mV, we assume that equilibration of membrane potential to the altered ion concentrations happens at least to a considerable extent as calculated by the GHK equation. Even if this would only reflect a part of the reality, the measured changes in membrane potential remain rather underestimated (see also Limitations of the Study) as studies using PMA reported a higher delta in membrane potential [11]. However, the smaller change in membrane potential could also be due to compensation mechanisms specific to our stimulus C5a. By using fMLP at a concentration of 10 *μ*M (which is reported to induce a maximal chemotactic response in neutrophils [46]), we demonstrated that the method is principally able to detect larger changes in membrane potential than evoked by C5a.

By using flow cytometry, we could avoid phagocytic triggering of the PMN by exposing the cells to devices such as patch-clamp electrodes. Furthermore, we avoided alteration in intracellular ion concentration and most importantly preserved cellular membrane integrity. Moreover, the applied method allowed measurement of thousands of cells per subject within seconds and supported the main finding (C5a-induced depolarization) by a high number (*n* = 45 for one minute of stimulation with C5a 100 ng/ml) of subjects. In addition, we demonstrated that our approach is not compromised by changes in cell shape or granularity (Figures 3(b)–3(e)). The method has proved to be applicable for translational studies, as it could define alterations of the transmembrane potential in a highly standardized and clinically most relevant hemorrhagic shock model.

### 4.3. Limitations of the Study

Measuring membrane potential by flow cytometry may have three downsides: First, the time resolution is limited, especially in comparison to patch-clamp techniques. We decided to start measuring after one minute of stimulation in order to enhance standardization and controllability of experiments. In addition, the method is limited by the properties of the used dye. The dye and/or the time resolution (and the issues discussed below regarding temperature) might be the reason why the results of this work differ quantitatively from other groups [9, 17, 47–49], as the reported results here are of lower magnitude. Second, membrane potential is proportionally linked to temperature, which is also represented within the GHK equation [37, 38]. While (pre)incubating cells, we stored the specimens at 37°C in a water bath. However, as cells are sucked into the flow cytometer, changes in surrounding temperature as well as mixing with rinsing fluid of the system are inevitable. Nonetheless, as temperature and—according to the GHK equation [37, 38]—therefore membrane potential are decreased by this effect, we might have rather underestimated instead of overestimated the changes in membrane potential. Third, we cannot measure absolute values of membrane potential, as we have no intracellular detector. Thus, we had to make several assumptions about intracellular concentrations and transcellular permeability of sodium and potassium. However, as literature findings in general agree that potassium conductance is in resting PMN most relevant [43, 44] compared to sodium conductance, we might have slightly miscalculated the membrane potential in absolute values. However, our estimate of the resting membrane potential at K4.5 with −65.4 mV is within the range of previous studies, which used an ionophore in order to obtain absolute values (−59 mV, with an additional hyperpolarization of 11 mV after addition of valinomycin [44], −73 mV [50], and −75 mV [10] and a patch-clamp-based approximation of the resting membrane potential of −50 to −60 mV [31]). Nevertheless, as we measured a calibration curve for every sample at every time point, the calculated change in membrane potential remains robust. While detection of the underlying alterations in defined ion channels being involved was beyond the scope of the present investigation, the changes of the membrane potential (in mV) validly reflects the overall alterations induced by the investigated conditions, irrespectively, whether it is invoked by sodium, potassium, or other ions. The following example not only demonstrates the limitation of the calculated values (Figure 1) but also strengthens the robustness of the measured change in membrane potential (Figure 2(a) and on): Assuming the conductance of sodium and potassium is 0.8 instead of 0.95 and 0.2 instead of 0.05, respectively, the calculated membrane potential would be −34.0 mV for K4.5 and −29.2 mV for K15. However, the calculated change in membrane potential for stimulated PMN with one minute of C5a 100 ng/ml remains comparable (3.0 mV with 0.8 potassium conductance versus 4.2 mV with 0.95 potassium conductance).

At last, we want to discuss the composition of the buffers. While most ions, glucose, pH, and osmolality were tightly controlled and adjusted to achieve physiological levels, the buffers lacked bicarbonate and consisted of an unphysiologically high chloride concentration. We accepted this limitation as bicarbonate, and consequently, buffers' pH is unstable in open systems, preferring precise pH control over normochloride solutions.

## 5. Conclusion and Outlook

Changes in transmembrane potential were a hallmark of C5a and fMLP, but not of C3a or LPS stimulation. Extracellular acidification enhanced the response while predepolarization and alkalinization diminished the response. Hemorrhagic shock almost completely suppressed the C5a response in membrane potential, which might represent a mechanism for development of neutrophil dysfunction in severe inflammation with coagulo- and complementopathy. Further research needs to verify these results preferentially with a different method and determine what extent of membrane potential change is helpful or harmful to PMN in order to exert their physiological functions.

In addition, exploration of the involved ion channels might reveal perpetrator ion channels that could be blocked to avoid development of immune incompetence of PMN and septic complications in shock.

## Figures and Tables

**Table 1:** Expression of CD88 on human PMN.

CD88	Mean	SD	
Upper SSC	0.0%	4.5%	ns
Lower SSC	−3.5%	4.3%	
Upper FSC	9.5%	4.0%	^∗∗^
Lower FSC	−9.1%	5.2%	
K15 versus Ctrl	−5.9%	5.3%	ns

Expression of CD88 after a 20-minute incubation with an antibody in Ctrl, respectively, K15 buffer. Upper and lower SSC/FSC demonstrate the dependency of C5aR expression of FSC, but not SSC (25% PMN each, *n* = 5). ^∗∗^ *p* < 0.01.

**Table 2:** Results of blood gas analyses of porcine hemorrhagic shock trial.

		Preshock		Postshock		Change
		Mean	SD	Mean	SD	Post/pre
Calculated membrane potential	**mv**	**−68.24**	**0.60**	**−66.53**	**1.60**	**−3%**
Leukocytes	/*μ*l	11511	3655	19892	5571	75%
K^+^	mM	3.30	0.22	4.06	0.88	23%
Na^+^	mM	140.80	0.45	140.20	3.56	0%
Ca^++^	mM	0.71	0.23	0.69	0.17	14%
Cl^−^	mM	95.20	2.86	98.80	0.84	4%
Anion gap	mM	26.76	3.12	25.70	2.25	−3%
pH		7.38	0.04	7.37	0.05	0%
pCO_2_	mmHg	39.54	4.46	35.42	4.69	−9%
pO_2_	mmHg	118.00	14.73	85.20	13.70	−26%
hb	g/dl	7.28	0.49	12.16	3.23	69%
hct	%	22.80	1.48	37.34	9.83	66%
sO_2_	%	99.04	0.75	95.14	2.71	−4%
Glucose	mg/dl	94.80	10.80	88.80	18.59	−7%
Lactate	mM	1.76	0.77	3.42	1.60	107%
Thrombocytes	/*μ*l	240800	71304	184600	60444	−23%

Hemorrhagic shock (*n* = 5) did not have a strong impact on calculated membrane potential, external ion concentration, and pH.

## Supplementary Materials

Supplementary Materials Supplement 1: equations 1–3 demonstrate the application of the GHK equation to calculate the change of membrane potential of PMN after stimulation.
