# Supplementary material for: Complement C5a Alters the Membrane Potential of Neutrophils during Hemorrhagic Shock
Source: Mediators Inflamm. 2018 May 29;2018:2052356. doi: 10.1155/2018/2052356 (PMC5996468; doi:10.1155/2018/2052356)
Supplement: Supplementary Materials — Supplement 1: equations 1–3 demonstrate the application of the GHK equation to calculate the change of membrane potential of PMN after stimulation. [file 2052356.f1.pdf]

Supplementary Materials description

Supplement 1: Equation 1-3 demonstrate the application of GHK equation to calculate the change of membrane potential of PMN after stimulation

Equation 1: simplified GHK equation to determine calculated membrane potential of PMN

$$\text{membrane potential [mV]} = -61\,\log\frac{P_K[K]_i + P_{Na}[Na]_i}{P_K[K]_e + P_{Na}[Na]_e}$$

Equation 2: change in fluorescence per change in membrane potential resulting in slope of calibration curve

$$\frac{\Delta\text{ fluorescence [AU]}}{\Delta\text{ 1 mV membrane potential}} = \frac{\text{fluorescence (K15} - \text{K4,5)}}{\text{membrane potential (K15} - \text{K4,5)}}$$

Equation 3: Calculation of change in membrane potential after stimulation

$$\Delta\text{ membrane potential} = \frac{\text{fluorescence (stimulant} - \text{control)}}{\text{slope}}$$
